# Ultrafast time-resolved extreme ultraviolet (XUV) photoelectron spectroscopy of hole transfer in a Zn/n-GaP Schottky junction

**DOI:** 10.1063/1.5046776

**Published:** 2018-10-22

**Authors:** Brett M. Marsh, Bethany R. Lamoureux, Stephen R. Leone

**Affiliations:** 1 Department of Chemistry, University of California, Berkeley, California 94720, USA; 2 Department of Chemistry, Purdue University, West Lafayette, Indiana 47907, USA; 3 Department of Physics, University of California, Berkeley, California 94720, USA; 4 Chemical Sciences Division, Lawrence Berkeley National Laboratory, Berkeley, California 94720, USA

## Abstract

The addition of a metal overlayer to a semiconductor photocatalyst is a frequently used synthetic route to passivate the surface and, via the formation of a Schottky barrier, to enhance catalytic activity of the photocatalyst material. While it is known that Schottky junctions decrease recombination by charge separation, measurements of the depletion region dynamics have remained elusive. Here, we use ultrafast pump-probe transient photoelectron spectroscopy to measure material-specific dynamics of the Zn/n-GaP(100) system. Through photoemission measurements the Schottky barrier height is determined to be 2.1 ± 0.1 eV at 10 monolayers of total Zn deposition. Transient photoemission measurements utilizing a 400 nm pump pulse show that, after excitation, holes are transferred from n-GaP(100) to the Zn overlayer within a few ps, as evidenced by shifts of the Zn 3d and Ga 3d core levels to higher binding energies. Within the timescale of the experiment (130 ps) no carrier recombination is observed in the junction. Furthermore, a long-lived surface photovoltage signal is observed at times >1 ms after photoexcitation. This work further exemplifies the potential of transient extreme ultraviolet photoelectron spectroscopy as a material-specific technique for the study of heterojunctions.

## I. INTRODUCTION

Gallium phosphide, a semiconducting material with an indirect band gap of 2.26 eV, has received much attention for its potential applications in optics, electronics, and photocatalysis.^1^ Of particular importance for photocatalysis is the ability of GaP to retain its original surface structure and electronic properties while operating in an aqueous solution. While the basic photocatalytic activity of untreated GaP is established, the already promising efficiency and stability of GaP can be improved through the use of coatings of metals or semiconductors.^2^ In the earliest work, Au and Ag films were deposited onto n- and p-type GaP wafers and the electrical properties were characterized in the presence and absence of illumination with light.^3^ The experiments showed a marked increase in photocurrent in these samples, as well as an increased resistance to corrosion in solution when compared to untreated GaP samples. Subsequent work focused on nanostructured GaP with a variety of dopant atoms, metals, and semiconductor materials on the surface, leading to further improvements in catalytic efficiency and stability.^2,4–6^

To understand the effect of surface treatments on the carrier lifetime and behavior in the depletion region of GaP junctions with other materials, the work here explores the Zn/n-GaP system using a high harmonic generation (HHG) based extreme ultraviolet photoelectron spectroscopy (XUV-PES) technique.^7^ In addition to the element, oxidation state, and surface sensitivity afforded by the photoemission method, time dependent dynamics are obtained by incorporation of a time-delayed UV/Vis laser pulse in conjunction with the XUV probe pulse. This technique has been previously used to observe the dynamics of electron transport in defect rich and defect poor TiO_2_ films on p-Si(100)^8^ as well as Zn layers on p-Si(100).^9^ Now, using the same technique, the dynamics of surface charging by hole transport from n-GaP to Zn are observed. Observation of the Ga 3d and Zn 3d core levels allows for the characterization of dynamics in both the GaP and Zn of the heterojunction in real-time.

In this work the barrier height as a function of Zn coverage is determined by monitoring the binding energy shift of the substrate Ga 3d as observed via XUV-PES. Then, through the use of transient XUV-PES, material-specific changes in the surface photovoltage of the Zn/n-GaP(100) system are observed in the overlayer (substrate) through energy shifts in the position of the Zn 3d (Ga 3d). The electronic properties, such as Fermi level pinning and carrier transport, which are observed and discussed in the transient XUV-PES measurements, serve to further the understanding of carrier dynamics within the depletion region of metal-semiconductor heterojunction photocatalytic systems.

## II. EXPERIMENTAL SETUP

The experimental apparatus used in these experiments has been previously described in detail.^7^ Briefly, the apparatus consists of a surface science chamber coupled to a laser and monochromator system that provides narrow band XUV femtosecond pulses for photoemission electron time-of-flight measurements. Ultrafast pulses are produced by a Spectra-Physics Spitfire amplifier producing 2.7 W average power of 35 fs pulses centered at 800 nm and 1 kHz repetition rate. The amplifier is seeded by a Spectra Physics Tsunami oscillator pumped by a 5 W frequency-doubled Nd:YVO4 continuous laser. The pulses from the amplifier are split into a probe beam, used to generate the XUV radiation, and a pump beam that is frequency doubled to 400 nm for excitation of the GaP semiconductor material.

The 800 nm probe pulses are focused into a semi-infinite gas cell at an intensity of 10^14^–10^15^ W cm^−2^. The cell is filled with Ar gas at approximately 25 Torr pressure. Under these conditions harmonics of the fundamental 800 nm light are produced from the 7th to 29th order, corresponding to 11 eV to 45 eV photon energy. These photon energies are sufficient to bring about photoemission from the 3d core levels of both Zn and Ga. The harmonics and residual 800 nm radiation impinge upon a plane grating after the gas cell where they are separated. By changing the angle of the grating, a single harmonic is selected to probe the sample. The selected harmonic is reflected by a cylindrical and toroidal mirror, resulting in a focused beam with a diameter of 0.2 mm. Any additional harmonics or the 800 nm fundamental are blocked by a slit at the entrance to the sample chamber.

The pump arm is directed to a variable delay stage after being split from the probe pulse to allow for time-resolved measurements. The 800 nm beam is passed through a beta-barium borate (BBO) crystal to produce 400 nm radiation by second harmonic generation. The resulting light is reflected from two 400 nm high reflector optics, removing the residual 800 nm radiation. The remaining light is focused by a lens and reflected onto the sample by a silver mirror located slightly above the XUV beam immediately before the sample chamber, resulting in a pump spot with 1 mm diameter at the sample.

The UHV end chamber is equipped with tools for preparation and characterization of surfaces. Among these are an Ar^+^ ion gun, used to clean the GaP(100) sample, a Zn oven, used to deposit the Zn film onto the GaP, and an Auger spectrometer for characterizing surface composition and coverage. Photoelectrons are collected and analyzed by a time-of-flight photoelectron spectrometer (TOF-PES) with a 1 m long double-walled *μ*-metal inner tube and a microchannel plate (MCP) detector at the end of the drift region. The signal is then acquired via a 5 GHz multichannel scaler unit.

The n-GaP(100) crystal used in this study is a bulk single crystal, grown by the Czochralski method, that was purchased from MTI corporation. Before Zn deposition, the n-GaP(100) film was cleaned using the argon gun with a filament emission current of 20 mA and a beam voltage of 3 kV for 10 min. Rounds of cleaning were repeated until an Auger electron spectroscopy spectrum of the bare n-GaP film presented only phosphorus and gallium peaks. Through atomic force microscopy imaging (supplementary material Fig. S1) the RMS roughness value was found to be 0.95 nm and 1.6 nm for an unsputtered and sputtered sample, respectively. The Zn films were grown by evaporation of zinc metal from a homebuilt evaporator. Briefly, the evaporator consists of a 4 cm long, 0.5 mm in diameter Ta filament wrapped around a piece of Zn metal. A current of approximately 2.80 A is applied to the Ta filament, which results in resistive heating of the filament and heating of the zinc. Due to the difference in Ta and Zn evaporation temperatures no Ta is observed on the sample after Zn deposition. Under these deposition conditions the Zn is found to deposit at a rate of 1.4 min/monolayer (see supplementary material Figs. 2 and 3). In this work no attempt is made at determination of the growth mechanism. Photoemission spectroscopy measurements of the junction system were recorded with the sample at a nominal temperature of 25 °C.

The sample is typically positioned 5 mm away from the entrance of the TOF-PES with the sample-surface-normal parallel to the spectrometer axis. This results in a laser beam incidence angle of 45° with respect to the surface normal. The sample position can be reproduced with an accuracy of 0.02 mm and 0.5°. The base pressure of the chamber is typically 2 × 10^−10 ^Torr and rises to 7 × 10^−10 ^Torr when the sample chamber is opened to the beamline, due to residual Ar gas from the HHG cell. The calibration of the energy scale of the TOF-PES is checked by acquiring photoelectron spectra at 3 adjacent harmonics, which are known to be spaced by 3.1 eV. Thus, the features in each PES are expected to also be spaced by 3.1 eV, giving an internal calibration standard. The angular acceptance of electrons in the TOF is 4° to either side of the surface normal, giving a total acceptance of 8°. Typical static photoemission spectra consist of an average of the collected photoemission spectra of 500 000 laser pulses. To efficiently acquire time-resolved data, each spectrum acquired in a transient measurement is composed of 180 000 laser pulses. In a transient PES experiment, time points between −5 to 5 ps are collected with 1 ps steps, while points outside this region are collected with approximately 20 ps steps. Cross correlation measurements of the pump and probe pulses by the laser assisted photoelectric effect (LAPE) gave an instrument response function of 80 fs for this experiment.

## III. SCHOTTKY JUNCTION: BARRIER HEIGHT CHARACTERIZATION

**FIG. 1.**
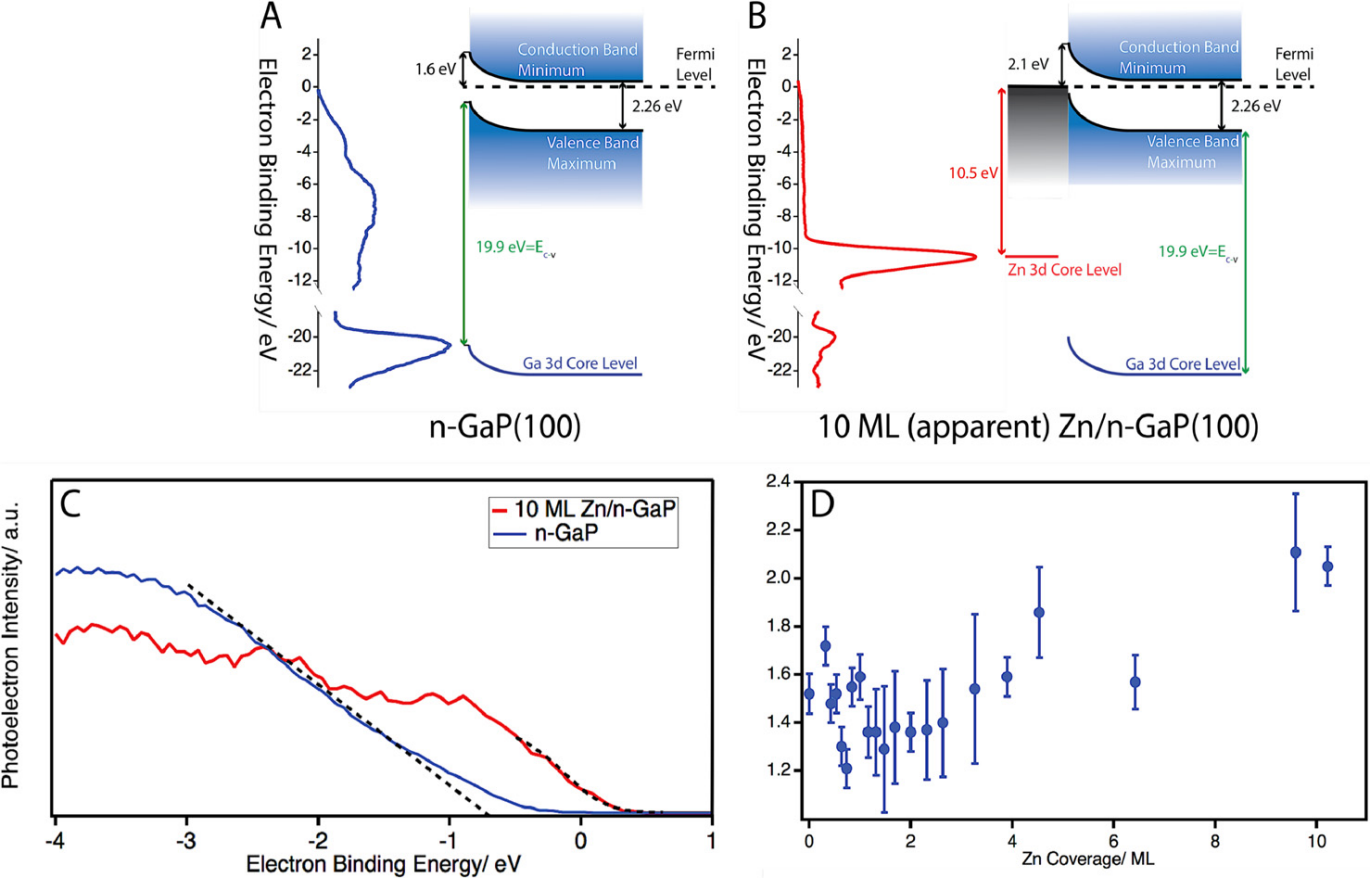
(a) Photoemission spectrum and band structure of n-GaP(100). (b) Photoemission spectrum and band structure of 10 ML Zn/n-GaP(100). (c) Enlarged view of the valence regions of n-GaP and 10 ML Zn/n-GaP. Dashed lines show the linear fit of n-GaP as well as the Fermi-Dirac fit for 10 ML Zn/n-GaP(100). (d) Measured barrier height as a function of Zn coverage.

The photoemission spectrum of clean n-GaP, recorded using the 27th harmonic of the nominal 800 nm fundamental from the Ti:Sapphire laser (41.9 eV photon energy), is shown in Fig. 1(a). To avoid any effects due to the generation of excited carriers by ambient light, all light sources in the chamber were turned off and all chamber viewports were covered during acquisition. All binding energies discussed herein are referenced to the center of a Fermi-Dirac distribution,^9,10^ which is fit to a spectrum of the tantalum clips holding the sample in place. For the Fermi-Dirac fit, the distribution is convoluted with a Gaussian function to account for instrumental broadening. The width of this Gaussian is only dependent on the instrument, and it was determined to be 0.5 eV by previous work in our group for this instrument.^9^

The n-GaP photoemission spectrum shows two distinct features [Fig. 1(a)]. The first, a broad and multi-peaked feature appears between −0.7 eV and −13.5 eV binding energy. The observed features are in good agreement with the previously calculated density of states for intrinsic GaP.^11^ The onset of this band, at 0.7 ± 0.1 eV below the Fermi level of the sample, indicates that the conduction band minimum is located 1.5 ± 0.1 eV above the Fermi level. This value is obtained by fitting to the zero-background rise of the valence band edge, with the error bars representing a single standard deviation. The onset of the valence band was found by a linear fit of the valence band edge. The value of the valence band offset is where the linear fit intercepts the x-axis [see Fig. 1(c)]. The second feature of interest, located at −20.7 ± 0.1 eV binding energy, corresponds to the Ga 3d core level. For this feature, the Ga 3d core level was fit with a Voigt line shape, with the error bars denoting one standard deviation. This peak location is in agreement with previous measurements of n-type GaP and other semiconductors incorporating Ga.^12–14^

The spectrum of 10 ML Zn deposited onto n-GaP is show in Fig. 1(b). Deposition of Zn induces several changes in the observed photoemission spectrum, with the foremost being the new, intense Zn 3d photoemission feature occurring at −10.6 ± 0.1 eV binding energy, as found by Voigt fitting. This feature is assigned to the 3d core level of the deposited Zn overlayer, as discussed in previous work from our group and others.^9,15^ While the Zn 3d core orbital of bulk Zn is composed of two spin-orbit split components of −10.2 and −10.3 eV, the resolution in our experiment is insufficient to observe this splitting. Finally, it is clear upon comparison of the clean n-GaP spectrum and the 10 ML Zn/n-GaP spectrum that the binding energy of the Ga 3d peak shifts from −20.6 ± 0.1 eV to −20.1 ± 0.1 eV. While the coverage of Zn greatly diminishes the Ga 3d signal due to the short mean free path of the Ga 3d electrons the peak is still visible. In a study of Zn deposition onto the GaP substrate (see supplementary material) it was observed that after forming a monolayer the Zn atoms form particles. Thus, the coverage is not uniform over the entire surface, allowing Ga 3d electrons to escape despite their short mean free path.

The shift of the Ga 3d core level is related to the change in band bending upon deposition of Zn and can be used to assess the Schottky barrier height, as outlined in several previous studies.^12,16,17^ Briefly, the energy difference between the valence band maximum of GaP and the Ga 3d core level is a constant value that is intrinsic to the semiconductor [denoted as E_c-v_ in Figs. 1(a) and 1(b)]. The Ga 3d binding energy, referenced to the Fermi level of the system, is known to shift with the bending of the valence and conduction bands. Since the Ga 3d shifts with the valence band, the changing position of the Ga 3d relative to the Fermi level, in conjunction with the known E_c-v_ value, gives the energetic separation of the valence band and the Fermi level. By subtracting this value from the band gap of GaP (2.26 eV),^18^ the separation of the Fermi level and conduction band can be found, thus giving the Schottky barrier height. The band diagrams for n-GaP(100) and 10 ML Zn/n-GaP(100) are shown in Figs. 1(a) and 1(b).

In Fig. 1(d) the measured Schottky barrier height as a function of Zn coverage is displayed. For these measurements photoelectron spectra were recorded sequentially at differing Zn coverages, followed by fitting as described above. At 0 ML of Zn, the barrier height of 1.5 ± 0.1 eV corresponds to the native n-GaP surface barrier to electron flow. As the coverage is increased, there is a clear shift of the barrier height to lower values, followed by a rise of the value with increasing Zn coverage. Such behavior has been previously observed for photoemission measurements of other metal-GaP contacts using synchrotron radiation, and it is explained by non-equilibrium effects that arise from the measurement techniques.^9,13,19^ Specifically, electron hole pairs are generated by interactions with photoelectrons leaving the material or by direct excitation of the substrate by the XUV radiation, which then segregate based upon the electric field present in the depletion region of the semiconductor. In this case, this means that holes are shuttled to the semiconductor surface, where they induce a long-lived photovoltage due to their slow recombination. The result is an initial shift of the Fermi level to higher binding energies at low surface coverages of Zn, giving a lower apparent barrier. This effect is also manifested as a shift of the Fermi level to a lower than expected value when compared to the reference (Ta sample holder) Fermi level. Such nonequilibrium effects are removed as the metal thickness on the semiconductor is increased, typically becoming negligible around 2 nm metal thickness.^13^ It should be noted that while this effect is similar to the phenomenon of surface charging in photoemission in non-metallic samples this effect arises purely from excitations of electron hole pairs during photoemission. In the case of these experiments, the measured barrier height at 10 ML (∼2.8 nm) of Zn coverage is used. This barrier height is found to be 2.1 ± 0.1 eV, indicating that Zn deposition induces a further 0.5 eV band bending in the n-GaP substrate, which is consistent with the formation of a Schottky barrier in this system.

The noise observed in these measurements is attributed to the relatively small signal of both the Ga 3d core level upon Zn deposition as well as the small signal of the Fermi level. The fits to these small features lead to increased errors in the fits used, thus giving the large error bars. Furthermore, while the position of the sample can be well reproduced, errors in the measured quantities may result from slight differences in the position or angle of the sample relative to the spectrometer.

## IV. TRANSIENT XUV-PES

**FIG. 2.**
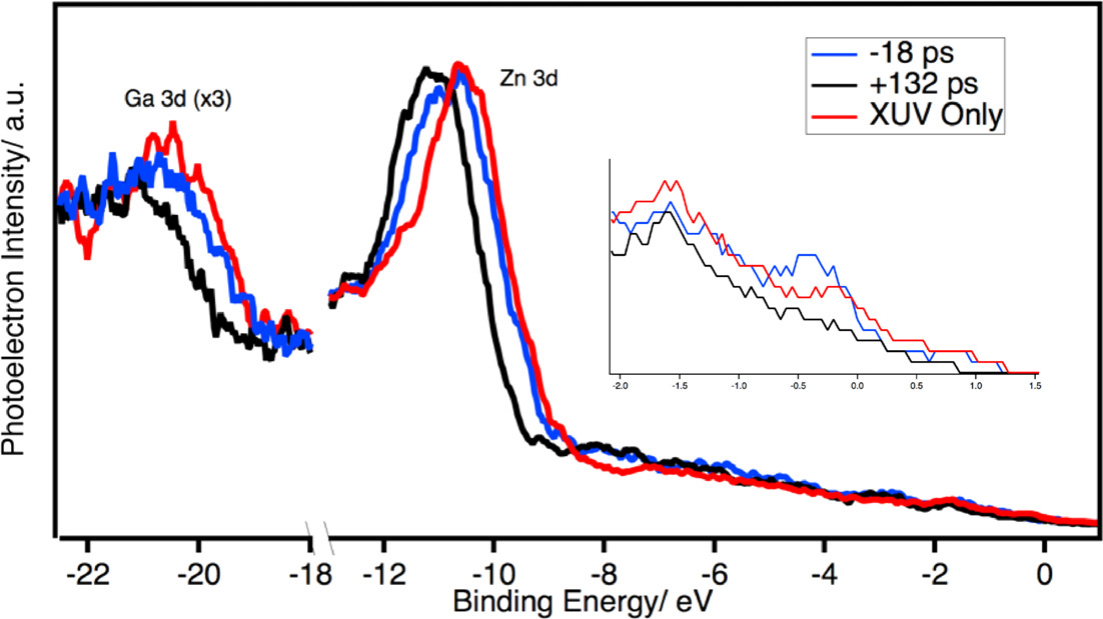
XUV only spectrum (red) compared to −18 ps (blue) and +132 ps (black) time delays for 10 ML Zn/n-GaP. The inset is an enlarged view of the Fermi level region of each trace.

To assess the behavior of Zn/n-GaP junction under 400 nm illumination, transient XUV-PES spectra are recorded for a series of pump-probe time delays. A negative time delay indicates that the probe beam precedes the 400 nm pump beam, while a positive time delay corresponds to excitation with the 400 nm pump beam before the XUV probe beam induces photoemission. The spectra of 10 ML Zn/n-GaP with XUV only, at −18 ps and +130 ps time delays are shown in Fig. 2 for a 400 nm excitation density of 2.5 mJ cm^−2^ (4.4 × 10^20^ carriers cm^−3^).^20^ While the carrier density was calculated assuming no attenuation by the 400 nm beam by the metal overlayer, studies have shown that as few as 10 nm layers of transition metals can attenuate the incoming radiation by 50%.^21,22^ This effect is dependent upon the metal geometry on the surface as well as the metal identity.^22^ However, even in the 50% attenuation case the number of carriers excited by the 400 nm radiation is still much larger than the doping concentration (10^18^ cm^−3^) of the substrate.

**FIG. 3.**
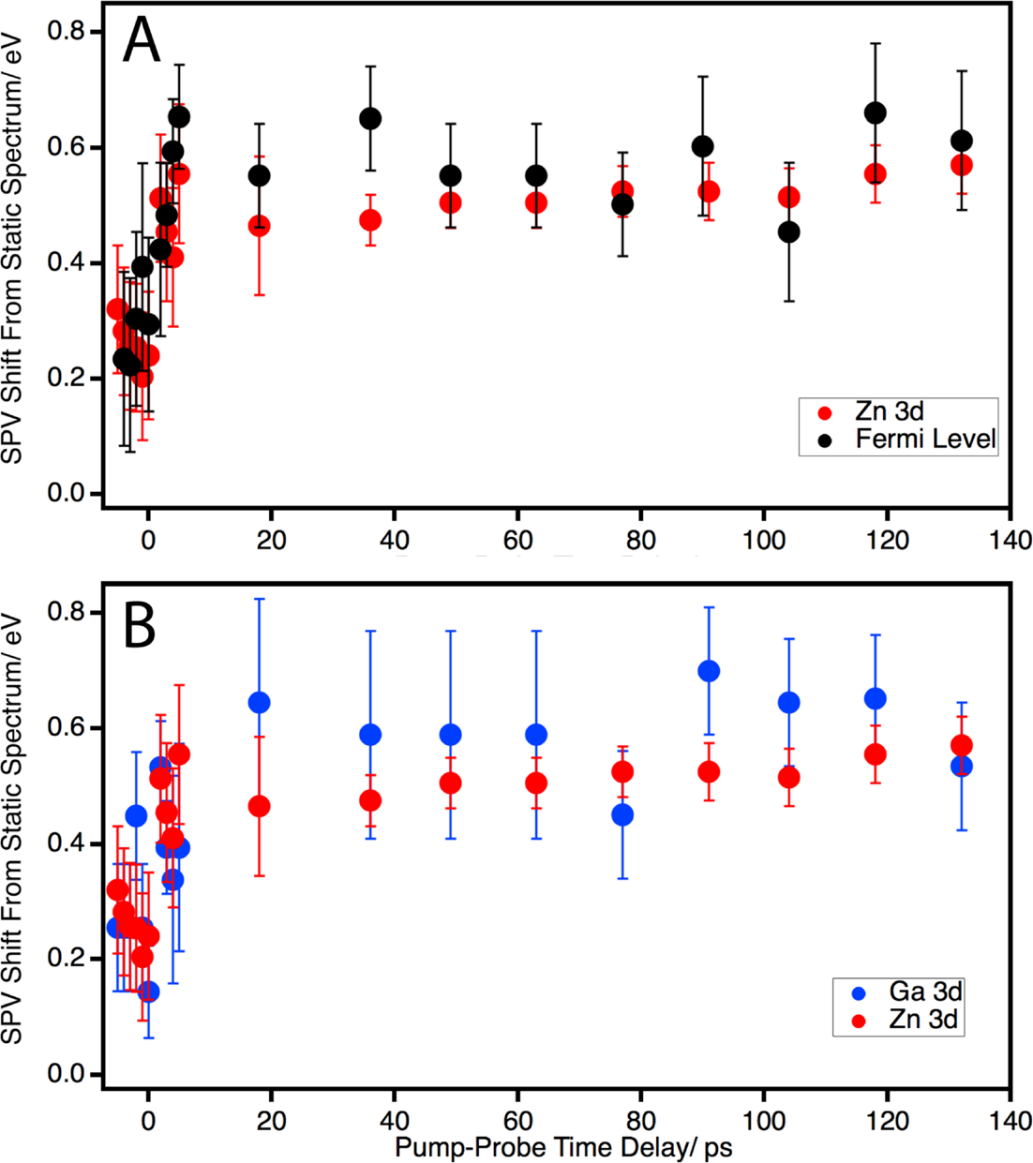
(a) Transient traces for the binding energy shift of the Zn 3d core level (red) and Fermi level (black). (b) Transient traces for the binding energy of the Zn 3d (red) and Ga 3d (blue) core levels.

For all the following discussion the features are fit as described in Sec. III. The difference between the −10 ps time delay and XUV only spectra are found to show a slight shift, indicating that the effect of the probe is minimal at negative (probe before pump) time delays. The origin of this shift is discussed in further detail below. However, at +130 ps there is a clear shift of the spectrum to higher binding energies. In the spectra there are three features of interest: the Zn 3d core level, Ga 3d core level, and the Fermi level, which consists of electrons from the Zn overlayer. For all three of these components a shift to higher binding energy is observed. The binding energy shift of these features as a function of the pump-probe time delay is shown in Fig. 3. A figure with additional negative timepoints is included in the supplementary material.

In Fig. 3(a) the transient behavior of the Zn 3d core level (red) and Fermi level (black) are shown. The Zn 3d and Ga 3d core levels were fit with a Voigt lineshape, while the Fermi level position was found by fitting with a Fermi-Dirac distribution.^9,10^ While the intensity of the Ga 3d peak is diminished under the conditions of the transient experiment the peak is still able to be observed and fit by a Voigt line shape. Examination of the data shows that the time scale of excitation is quite similar for both features. In addition to the similar time-scales, the absolute magnitudes of the Zn 3d and Fermi level shifts for both features are similar, with a shift of approximately 0.6 eV towards higher binding energy compared to the static XUV only spectrum. In Fig. 3(b) the transient behavior of the Ga 3d (blue) is shown in comparison to the Zn 3d (red). The Ga 3d core level shifts with a similar magnitude and time to that of the Zn 3d core level and the Fermi level. This indicates the dominant dynamic processes within the Zn overlayer and depletion region within GaP are similar. In work performed by Kamada and coworkers on a Cs/p-GaAs junction under continuous illumination, a similar trend was observed, with both Cs and Ga core levels shifting by a similar magnitude and sign compared to the unilluminated case.^23^

**FIG. 4.**
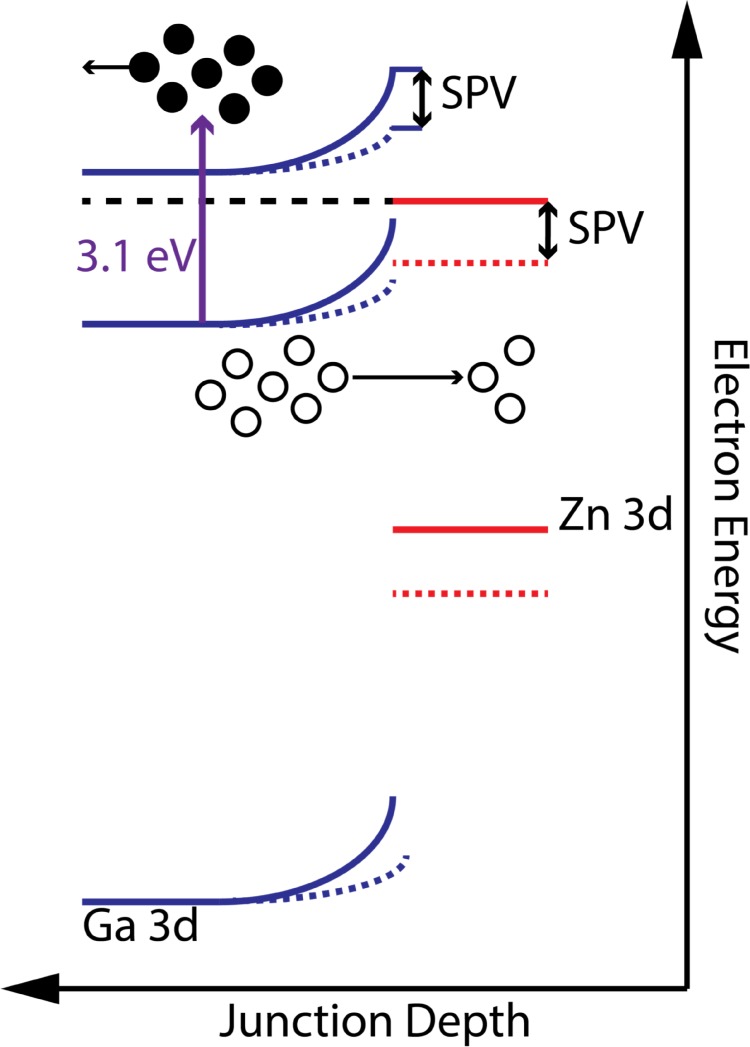
Band diagram of 10 ML Zn/n-GaP in the absence (solid lines) and presence (dotted lines) of the 400 nm pump beam. Photoexcited electrons are given as filled circles, while photogenerated holes are represented by empty circles. Hole transport to the surface results in an apparent increase in electron binding energy. Electron energy denotes the energy of electrons in the junction.

To understand the processes responsible for the observed shifts, the behavior of the Zn 3d and Ga 3d core levels will be considered first. It is clear from the similarity of their dynamics that the process that modulates the Zn 3d binding energy is also most likely responsible for the Ga 3d shift. The space charge region of the material is negatively charged due to the n-type doping of the substrate. Thus, holes are drawn towards the surface while electrons are shuttled into the bulk of the sample. The net result is a lowering of the electron quasi-fermi level in the Zn film and depletion region of n-GaP, which also reduces the band bending in n-GaP. While electron accumulation occurs in the bulk of n-GaP, the probe depth of XUV-PES is only a few nanometers, meaning that the results are mainly sensitive to the depletion region of n-GaP. Thus, the dynamics observed are assigned to the trapping of holes in the Zn layer, which also screens the electric field in the n-GaP depletion region, decreasing the band bending. A schematic picture of this process is shown in Fig. 4.

In the case of carrier transport from the semiconductor to the surface layer it is expected that the Zn 3d and Zn Fermi level should show similar dynamics, as observed in a transient XUV-PES experiment from this laboratory on a 3.5 ML Zn/p-Si(100) junction.^9^ It was observed in that work that for excitation densities in which band bending in p-Si(100) was the limiting factor, the Zn 3d and Fermi level shifted by a similar magnitude, while for higher excitation densities the Zn 3d showed a larger shift than the Fermi level. In both cases, the two features showed a similar time dependence. With this knowledge, along with the 0.6 eV shift in binding energy, which is well below the calculated band bending of 1.2 eV in the Zn/n-GaP junction, the dynamics observed here support the assignment of the dynamics as being induced by the transport of holes to the Zn layer after excitation, followed by a reduction in band bending throughout the junction. It is clear from the collected data that the SPV signal shows no change through the end of the delay ranged scanned, and thus no attempt is made to assign a timescale for recombination. While a transient rise is observed around time zero there is a 0.2 eV background shift when compared to an unpumped photoelectron spectrum. Due to this background, which is discussed below, the timescale for this initial rise is not assigned.

It is important to note that the *pump*-induced SPV discussed above is fundamentally the same as the previous discussed *probe*-induced SPV which influences the observed Schottky barrier heights (Sec. III). However, probe-induced SPV is only observed in the case in which the substrate is strongly excited by the probe radiation. Here, the thickness of the Zn overlayer was chosen to minimize probe-induced SPV in our experiments as discussed in Sec. III.

Finally, as noted above, there is a clear offset of the photoemission spectrum at negative time delays relative to the XUV only spectrum of approximately 0.25 eV for all features. There are two possible explanations for this behavior: pump space-charge effects and long-lived states excited by the 400 nm pump beam. Pump space charge effects can arise from the interaction of photoelectrons induced by multiphoton absorption of the 400 nm pump beam and the XUV probe induced photoelectrons.^24,25^ In these experiments, the total number of photoelectrons induced by the 400 nm pump beam is kept below 0.05 electrons per pulse through the time of flight to avoid these effects. For reference, the number of photoelectrons induced by the XUV beam, which is the lowest stable number of counts that can be obtained using this HHG source, is on the order of 0.2 electrons per pulse. Although the number of observed electrons originating from the pump beam is 0.05 per pulse, the lowest energy electrons may not be observed at the detector due to stray electric fields, which can influence their flight.

While pump-induced space charge effects can have a strong influence on the observed transient spectrum, at negative times it is expected that the photoemission spectrum would shift to *lower* binding energies in the presence of space charge.^24^ The observed shift in this experiment at negative times is to higher binding energy, thus pump-induced space charge effects are not consistent with the observed data. Furthermore, it is expected that pump-induced space charge effects will lead to a rising increase in the surface photovoltage shift before time zero over a period of 100 ps. In these experiments the surface photovoltage before time zero is essentially constant over this time frame, again indicating that pump-induced space charge effects are not influencing the phenomena observed here.

The second possibility, a long-lived surface photovoltage resulting from the 400 nm pump beam, is the more likely cause of this persistent shift, as explained next. In our experiment, we observe that the SPV persists between probe pulses, which are spaced 1 ms apart at a 1 kHz repetition rate, implying a multiple millisecond decay of the SPV in the Zn/n-GaP system. While previous studies of GaAs semiconductors^23,26^ and p-Si(100) junctions suggest that surface photovoltage decays in these systems on the picosecond timescale, it is valuable to consider the difference in band gaps between the different materials to get a clearer picture of the dynamics occurring. For example, in the case of Zn on p-Si(100), the band gap of silicon is 1.12 eV, and the Schottky barrier height was found to be 0.725 eV. However, n-GaP has a band gap of 2.26 eV, and the Zn/n-GaP barrier height is 2.1 eV. It is known that recombination in Schottky junction systems is governed by a modified thermionic emission law known as the Schottky formalism

$$J=AT^{2}e^{-\beta (\phi -SPV)},$$

where *A* is the Richardson constant, *β* is 1/*k_B_T*, *φ* is the Schottky barrier height, and SPV is the measured surface photovoltage.^27^ For similar Richardson constants and temperatures, it is clear that the main factor determining the recombination rate is the barrier height. Since a wide band gap semiconductor will likely have a higher barrier,^17^ it is then inferred that the recombination rate should be significantly decreased for systems in which the semiconductor has a wide band gap. For the wide band gap semiconductors ZnO and TiO_2_, surface photovoltages have been observed to persist for milliseconds to seconds, depending on surface conditions.^28–30^ It should also be noted that as the SPV decays, the difference between Schottky barrier height and SPV becomes larger, resulting in a lowering of the recombination rate over the barrier, limiting the decay rate. This is suggested as the origin of the long-lived SPV observed in these experiments.

It should be noted that previous studies have indicated that the interaction between an electron leaving the surface and the electric field generated by SPV may have an effect on the observed transient spectra.^31^ Specifically, the observed dynamics may show a SPV present at negative time delays, as observed in the data here. However, this effect will also cause a slow rise of the photovoltage shift at negative time delays, which does not occur in the data. Thus, it is unlikely that a time-evolving electric field effect in the sample is the origin of the observed shift. The characterized transients relative to this shift are the only processes reported here.

## V. CONCLUSIONS

In this work the photoexcitation dynamics of a 10 ML Zn/n-GaP(100) system were measured using a HHG based transient XUV-PES technique. XUV-PES spectra reveal that deposition of Zn results in an additional 0.5 eV of band bending from the clean n-GaP surface, giving a Schottky barrier height of 2.1 eV and showed a 1.5 eV surface barrier height in clean n-GaP. Transient photoemission measurements, using 400 nm pump pulses, show that the Zn and Ga 3d core levels and Zn Fermi level shift by approximately 0.6 eV to higher binding energy and display similar dynamics. Such results are indicative of hole transport from n-GaP into the Zn overlayer, resulting in a reduction in band bending at the interface and a shift of the electron quasi-Fermi level to lower energy. These findings contrast with the results obtained for studies of p-type Si with Zn in our group, in which electron transport to the Zn layer was the dominant process.^9^ The observed dynamics also indicate that there is a long-lived population of holes in the Zn overlayer, persisting for over 1 ms after the initial excitation. This population is attributed to the slowing of the thermionic emission process as the Schottky barrier is recovered after excitation. Here, element-specific photoelectron signals are derived not only from the deposited surface material, but also from the underlying semiconductor elements in the junction. Therefore, this work shows the powerful possibilities for element and material-specific XUV-PES measurements at junctions, providing for future measurement of a wide number of heterojunction systems with photoelectron spectroscopy on femtosecond and picosecond timescales.

Finally, the results of this study show both the promise and problems with time-resolved photoemission at short timescales. While it is clear that the electronic structure of the sample can be probed in great detail using XUV photoemission, and that short timescale dynamics are well captured, the long timescales associated with electron-hole recombination in this junction (and likely in other wide-bandgap semiconductor-metal junctions) are not adequately measured. This issue is due to the repetition rate of the laser, which in this experiment is 1 kHz, although it can be much higher in other tabletop laser-based experiments, and due to the physical limitations of delay stages. While femtosecond experiments have had success with narrow bandgap semiconductor systems, it is clear that approaches with longer timescales, such as synchrotron-based experiments, may be beneficial for systems with much longer lifetimes. Experiments with nanosecond laser systems with repetition rates of 10–20 Hz may also prove useful when coupled to an XUV source such as a helium lamp.

## SUPPLEMENTARY MATERIAL

See supplementary material contains AFM images of the sputtered and unsputtered surface and Auger Electron Spectroscopy data for the GaP/Zn depositions. An extended transient showing negative time points is also included.
